# The role of blood pressure in risk of ischemic and hemorrhagic stroke in type 1 diabetes

**DOI:** 10.1186/s12933-019-0891-4

**Published:** 2019-07-09

**Authors:** Stefanie Hägg-Holmberg, Emma H. Dahlström, Carol M. Forsblom, Valma Harjutsalo, Ron Liebkind, Jukka Putaala, Turgut Tatlisumak, Per-Henrik Groop, Lena M. Thorn, the FinnDiane Study Group

**Affiliations:** 1 Folkhälsan Institute of Genetics, Folkhälsan Research Center, Biomedicum Helsinki, Haartmaninkatu 8, 00290 Helsinki, Finland; 2 Abdominal Center Nephrology, University of Helsinki and Helsinki University Hospital, Helsinki, Finland; 3 Research Program for Clinical and Molecular Metabolism, Faculty of Medicine, University of Helsinki, Helsinki, Finland; 4 Department of Neurology, Helsinki University Hospital, Helsinki, Finland; 5 Department of Clinical Neuroscience/Neurology, Institute of Neuroscience and Physiology, Sahlgrenska Academy at University of Gothenburg, Göteborg, Sweden; 6 Department of Neurology, Sahlgrenska University Hospital, Göteborg, Sweden; 7 Department of Diabetes, Central Clinical School, Monash University, Melbourne, VIC Australia; 8 Department of General Practice and Primary Health Care, University of Helsinki, Helsinki, Finland

**Keywords:** Blood pressure, Cerebrovascular disease, Hemorrhagic stroke, Hypertension, Ischemic stroke, Type 1 diabetes

## Abstract

**Background:**

Hypertension is one of the strongest risk factors for stroke in the general population, while systolic blood pressure has been shown to independently increase the risk of stroke in type 1 diabetes. The aim of this study was to elucidate the association between different blood pressure variables and risk of stroke in type 1 diabetes, and to explore potential nonlinearity of this relationship.

**Methods:**

We included 4105 individuals with type 1 diabetes without stroke at baseline, participating in the nationwide Finnish Diabetic Nephropathy Study. Mean age at baseline was 37.4 ± 11.9 years, median duration of diabetes 20.9 (interquartile range 11.5–30.4) years, and 52% were men. Office systolic blood pressure (SBP) and diastolic blood pressure (DBP) were measured. Based on these pulse pressure (PP) and mean arterial pressure (MAP) were calculated. Strokes were classified based on medical and autopsy records, as well as neuroimaging. Cox proportional hazard models were performed to study how the different blood pressure variables affected the risk of stroke and its subtypes.

**Results:**

During median follow-up time of 11.9 (9.21–13.9) years, 202 (5%) individuals suffered an incident stroke; 145 (72%) were ischemic and 57 (28%) hemorrhagic. SBP, DBP, PP, and MAP all independently increased the risk of any stroke. SBP, PP, and MAP increased the risk of ischemic stroke, while SBP, DBP, and MAP increased the risk of hemorrhagic stroke. SBP was strongly associated with stroke with a hazard ratio of 1.20 (1.11–1.29)/10 mmHg. When variables were modeled using restricted cubic splines, the risk of stroke increased linearly for SBP, MAP, and PP, and non-linearly for DBP.

**Conclusions:**

The different blood pressure variables are all independently associated with increased risk of stroke in individuals with type 1 diabetes. The risk of stroke, ischemic stroke, and hemorrhagic stroke increases linearly at blood pressure levels less than the current recommended treatment guidelines.

**Electronic supplementary material:**

The online version of this article (10.1186/s12933-019-0891-4) contains supplementary material, which is available to authorized users.

## Background

Hypertension is one of the strongest risk factors for stroke in the general population, and the risk of stroke increases linearly when blood pressure levels exceed 115/75 mmHg [1]. Blood pressure variables, such as systolic blood pressure (SBP), diastolic blood pressure (DBP), and mean arterial pressure (MAP), are all associated with an increased risk of stroke [2], and lowering of the blood pressure with any antihypertensive agent reduces the risk with as much as 30% [3]. However, while lowering blood pressure levels linearly decreases the risk of stroke in the general population, there has been evidence of a J-shaped phenomenon in individuals with diabetes [4, 5]. Zhao et al. showed that a systolic blood pressure < 110 mmHg, as well as > 160 mmHg, significantly increases the risk of stroke in individuals with diabetes. This J-shaped association is significant specifically in younger individuals under the age of 60.

Blood pressure, SBP especially, has been identified as an important risk factor for stroke also in type 1 diabetes [6, 7]. We have also previously shown that systolic blood pressure, among longer diabetes duration, diabetic micro- and macrovascular complications, poor glycemic control, and a history of smoking, is independently associated with an increased risk of stroke in type 1 diabetes [8]. How the other blood pressure variables DBP, MAP and PP are associated with the risk of stroke and its subtypes is still unclear in type 1 diabetes. Increased sodium intake, expressed as a higher urinary sodium excretion, leads to higher blood pressure, and to increased risk of cardiovascular disease, stroke included [9]. Paradoxically, in individuals with type 2 diabetes, lower 24-h urinary sodium (24-h Na) excretion increased the risk of all-cause and cardiovascular mortality [10]. The same was seen in individuals with type 1 diabetes even after adjusting for hypertension [11]. While higher sodium intake increases the cardiovascular risk in the general population, higher potassium consumption seems to have a protective influence on this risk [12]. It is not known how sodium and potassium excretion affect the risk of stroke in type 1 diabetes, nor is it known how low blood pressure levels affect the risk of stroke in these individuals.

The aim of this study was to elucidate the impact of different blood pressure variables and levels on the risk of stroke and its subtypes ischemic stroke and hemorrhagic stroke in individuals with type 1 diabetes. Moreover, we wanted to determine the impact of urinary sodium and potassium excretion on the risk of stroke.

## Methods

All participants were part of the Finnish Diabetic Nephropathy (FinnDiane) Study, a nationwide multicenter study with the aim to uncover risk factors and mechanisms for micro- and macrovascular complications of type 1 diabetes. The study was launched in 1997, and patient recruitment is still ongoing. A detailed description of the research design and the population has previously been reported [13]. All adult individuals with type 1 diabetes, attending the 77 participating study centers’ (Additional file 6: Appendix) diabetes and/or renal outpatient clinics, were consecutively asked to participate in the study. The participants in the study underwent a clinical examination at a regular visit to the attending physician. Both the attending physician, as well as the participant, completed standardized questionnaires regarding the participant’s medical condition, medical history and medication, as well as smoking habits. In addition, blood samples were drawn, and overnight or 24-h urine collections were performed. Follow-up information is collected regularly from registries and review of medical files. For this study, we excluded participants without information on systolic blood pressure (151 participants), diastolic blood pressure (two participants), and without information on antihypertensive medication (32 participants). Due to unclear information on stroke at baseline, a total of 15 individuals with stroke were excluded from the study. In addition, two participants were excluded due to subdural hemorrhages, one due to traumatic hemorrhage, one due to perinatal hemorrhage, and one due to hypertensive encephalopathy. This resulted in a total of 4105 individuals with type 1 diabetes in the FinnDiane database without a stroke at baseline and with complete information on stroke during follow-up available. The local ethics committee of each center approved the study protocol, and the study was carried out in accordance with the Declaration of Helsinki. Each participant signed a written informed consent.

### Diabetes and renal status

We defined type 1 diabetes as diabetes diagnosis before 40 years of age and insulin medication commenced within 1 year after diagnosis. The mean age at baseline was 37.4 ± (standard deviation) 11.9 years, the median duration of diabetes was 20.9 (interquartile range 11.5–30.4) years, and 52% of the participants were men. Each participant collected timed urine samples for the measurement of urinary albumin excretion rate (UAER). Diabetic nephropathy (DN) was defined as having a UAER of ≥ 200 µg/min or ≥ 300 mg/24 h or having end-stage renal disease (ESRD). ESRD was defined as ongoing dialysis treatment or kidney transplantation. Severe diabetic retinopathy (SDR) was defined as history of retinal photocoagulation. Coronary heart disease (CHD) was defined as a history of myocardial infarction or coronary artery revascularization, or treatment with long-acting nitroglycerin.

### Blood pressure, sodium, and potassium measurements

Blood pressure was measured by a trained nurse during the exam visit twice in the sitting position with a 10 min rest before the first measurement, and the mean values of these two measurements were calculated for both the systolic blood pressure (SBP) and the diastolic blood pressure (DBP). Pulse pressure (PP) was calculated as SBP–DBP. Mean arterial pressure (MAP) was calculated as $${\text{DBP}} + {\raise0.7ex\hbox{$1$} \!\mathord{\left/ {\vphantom {1 3}}\right.\kern-0pt} \!\lower0.7ex\hbox{$3$}}{\text{PP}}$$. Antihypertensive medication was defined as the use of any anti-hypertensive agent; angiotensin-converting-enzyme inhibitors, angiotensin receptor blockers, calcium channel blockers, β-blockers, diuretics, or other antihypertensive agents (mainly moxonidine). Hypertension was defined as SBP > 140 mmHg and/or DBP > 90 mmHg. 24-h Na and 24-h urinary potassium (24-h K) excretion were measured from a 24-h urine collection. Sodium/potassium ratio (Na/K ratio) was defined as 24-h Na divided by 24-h K. Information on sodium and potassium urinary excretion was available for 2402 (59%) individuals during follow-up.

### Stroke

A detailed description of the identification and classification of the strokes has previously been described in detail [13]. For this study, we had additional follow-up data available until the end of 2012. Follow-up data was available in all participants. In short, individuals with an incident stroke were identified from the FinnDiane questionnaires, death certificates, and the National Care Register of Health Care. Medical records from the attending hospitals, brain computed tomography and magnetic resonance images, as well as death certificates were ordered on these individuals. Based on these data, all the strokes were classified into ischemic or hemorrhagic stroke by two stroke neurologist (J.P and R.L), with the help from an experienced neuroradiologist when needed. Follow-up time was calculated from the baseline visit until the last date the participants were known to be free of stroke, or until the date of the first stroke for those who suffered a stroke, resulting in 45,050 person-years of follow-up.

### Statistical analyses

All variables included were tested for normal distribution by visual inspection. Parametric continuous variables were analyzed with Student’s t-test, and the results are presented as means with standard deviation. Non-parametric variables were analyzed with Mann–Whitney U-test, the results are presented as medians with interquartile range. The difference in categorical variables between groups was tested with the χ^2^-test. Cox proportional hazard models were performed to study how SBP, DBP, MAP, and PP affected the risk of any stroke, ischemic stroke, and hemorrhagic stroke. The results are presented as hazard ratio (HR) with 95% confidence interval (CI). Blood pressure variables were included in different models because of collinearity, with only one blood pressure variable in each model. The multivariate models were adjusted for the variables that statistically differed in the univariate analyses (Additional file 2: Table S2), which resulted in adjustments for sex, diabetes duration, waist circumference, DN, SDR, HbA_1c_, LDL-cholesterol, current or history of smoking, use of antihypertensive medication, presence of CHD, and year of the FinnDiane study visit.

Since participant mortality due to other causes than stroke is a competing event to stroke, we performed competing risk analyses to take this into account. We examined the association between SBP and stroke using the proportional subdistribution hazards regression model by Fine and Gray [14], with death as a competing event. The results are presented as subhazard ratio (SHR) with 95% CI, and is defined as the hazard of a given cause in the presence of the competing event.

To assess the shapes of the association between the continuous variables SBP, DBP, MAP, and PP and the risk of stroke, we fitted unadjusted Cox proportional hazard models with each variable included as a restricted cubic spline with three knots. The number of knots was selected based on the Akaike information criteria [15], and located at the 10th, 50th, and 90th percentiles, corresponding to the values listed in Additional file 1: Table S1. The median values were used as the reference point in the analyses. HR was then estimated based on the restricted cubic spline models and used to create plots for graphical assessment of the relationships. We implemented the Wald test to determine whether the relationship between the blood pressure variables and stroke significantly deviated from linearity. P < 0.05 was considered statistically significant. The restricted cubic spline analyses were performed with Harrell’s Regression Modelling Strategies (rms) package [16], while the competing risk analyses were performed with the ‘cmprsk’ package [17] in the R software [18]. All other analyses were performed with the SPSS Statistical software 25.0 (IBM Corporation, Armonk, NY).

## Results

In total, 202 (5%) individuals with type 1 diabetes suffered an incident stroke during follow-up. Of these, 145 (72%) individuals suffered an ischemic stroke, and 57 (28%) a hemorrhagic stroke. The blood pressure variables, antihypertensive medication, and urinary sodium and potassium excretion of those with an incident stroke compared to those without stroke during follow-up are shown in Table 1. All measures of blood pressure levels were higher in those with an incident stroke. The use of any type of antihypertensive medication was far more common in those with a stroke, and no differences in sodium or potassium excretion were seen. The other characteristics of those with an incident stroke compared to those without are shown in Additional file 2: Table S2. In short, those with a stroke were older, had a longer duration of diabetes and more cardiovascular complications. In addition, DN, SDR, and smoking were more frequently occurring in those with an incident stroke. Those with stroke had higher total cholesterol, LDL-cholesterol, and triglycerides, as well as poorer glycemic control.

**Table 1:** Clinical characteristics according to type of stroke during follow-up

Baseline data	No stroke	Any stroke	Ischemic stroke	Hemorrhagic stroke
*n*	3903	202	145	57
Men (%)	51	63*	63*	61
Age (years)	36.9 ± 11.8	45.8 ± 9.5*	46.6 ± 9.5*	44.4 ± 9.1*
Duration of diabetes (years)	20.0 (11.7–29.3)	31.0 (24.4–36.8)*	32.0 (25.4–37.0)*	28.6 (25.0–34.9)*
Age at stroke (years)	–	52.2 ± 9.8	53.1 ± 10.0	49.9 ± 8.9
Blood pressure measurements				
Systolic blood pressure (mmHg)	133 ± 18	150 ± 23*	150 ± 23*	149 ± 23*
Diastolic blood pressure (mmHg)	79 ± 10	83 ± 11*	83 ± 11*	83 ± 12*
Pulse pressure (mmHg)	54 ± 16	67 ± 19*	68 ± 20*	66 ± 18*
Mean arterial pressure (mmHg)	97 ± 11	105 ± 13	105 ± 13*	105 ± 14*
Hypertensive (%)	32	66*	68*	60*
24-h urinary sodium excretion (mmol/l)	140 (101–185)	145 (98–192)	149 (98–202)	138 (96–161)
24-h urinary potassium excretion (mmol/l)	83 (62–105)	79 (59–104)	79 (56–104)	82 (61–103)
Sodium/potassium ratio	1.74 (1.32–2.30)	1.76 (1.45–2.33)	1.81 (1.47–2.35)	1.67 (1.39–1.78)
Medication				
Antihypertensive medication (%)	36	78*	78*	77*
ACE inhibitor (%)	24	45*	48*	38*
Angiotensin receptor blocker (%)	5	12*	12*	11
Calcium channel blocker (%)	11	33*	35*	27*
β-Blocker (%)	12	38*	38*	38*
Diuretic (%)	11	36*	36*	38*
Other (%)	1	2*	1	4*

Data are presented as mean ± standard deviation, median with interquartile range, or number of cases (%)

*ACE inhibitor* angiotensin-converting-enzyme inhibitor

*P < 0.05 compared to no stroke

Cox proportional hazards models were performed to study the independent effect of blood pressure variables on the risk of stroke; the results are shown in Table 2. For any stroke, SBP, DBP, MAP, and PP all independently increased the risk of stroke. For ischemic stroke, SBP, MAP, and PP independently increased the risk, while DBP was not significant in the model (Table 2). For hemorrhagic stroke, SBP, DBP, and MAP independently increased the risk, while PP was not significant (Table 2). When DBP, MAP, and PP, in turn, were included in the same model as SBP, SBP remained significant in the model for any stroke and ischemic stroke, while for hemorrhagic stroke, only MAP remained significant in the model (data not shown). Taking death into consideration as a competing event to stroke, the SHR for the different blood pressure variables did not significantly change for any type of stroke or ischemic stroke. For hemorrhagic stroke, DBP was no longer significant in the analyses (Table 2).

**Table 2:** Blood pressure as a risk factor for any stroke, ischemic stroke, and hemorrhagic stroke

	Any stroke (HR)	P	Any stroke (SHR)	P
SBP, per 10 mmHg	1.20 (1.11–1.29)	< 0.001	1.17 (1.09–1.25)	< 0.001
DBP, per 10 mmHg	1.21 (1.03–1.42)	0.021	1.19 (1.02–1.37)	0.026
MAP, per 10 mmHg	1.32 (1.16–1.52)	< 0.001	1.27 (1.12–1.42)	< 0.001
PP, per 10 mmHg	1.19 (1.10–1.30)	< 0.001	1.16 (1.07–1.25)	< 0.001

	Ischemic stroke (HR)	P	Ischemic stroke (SHR)	P
SBP, per 10 mmHg	1.19 (1.09–1.30)	< 0.001	1.16 (1.07–1.25)	< 0.001
DBP, per 10 mmHg	1.15 (0.95–1.39)	0.148	1.13 (0.94–1.33)	0.170
MAP, per 10 mmHg	1.29 (1.10–1.51)	0.002	1.23 (1.06–1.40)	0.007
PP, per 10 mmHg	1.21 (1.09–1.33)	< 0.001	1.17 (1.06–1.27)	0.002

	Hemorrhagic stroke (HR)	P	Hemorrhagic stroke (SHR)	
SBP, per 10 mmHg	1.21 (1.05–1.41)	0.010	1.16 (1.02–1.31)	0.023
DBP, per 10 mmHg	1.38 (1.01–1.87)	0.040	1.31 (0.98–1.65)	0.064
MAP, per 10 mmHg	1.42 (1.10–1.85)	0.008	1.32 (1.04–1.59)	0.024
PP, per 10 mmHg	1.16 (0.98–1.38)	0.089	1.13 (0.98–1.27)	0.089

Data are presented as hazard ratio with 95% confidence interval, as well as subhazard ratio with 95% confidence interval. Model also included sex, waist circumference, diabetes duration, diabetic nephropathy, HbA_1c_, LDL-cholesterol, severe diabetic retinopathy, any smoking, antihypertensive medication, coronary heart disease, and year of the FinnDiane study visit. Blood pressure variables were included in separate models

*SBP* systolic blood pressure, *DBP* diastolic blood pressure, *MAP* mean arterial pressure, *PP* pulse pressure

**Fig. 1:**
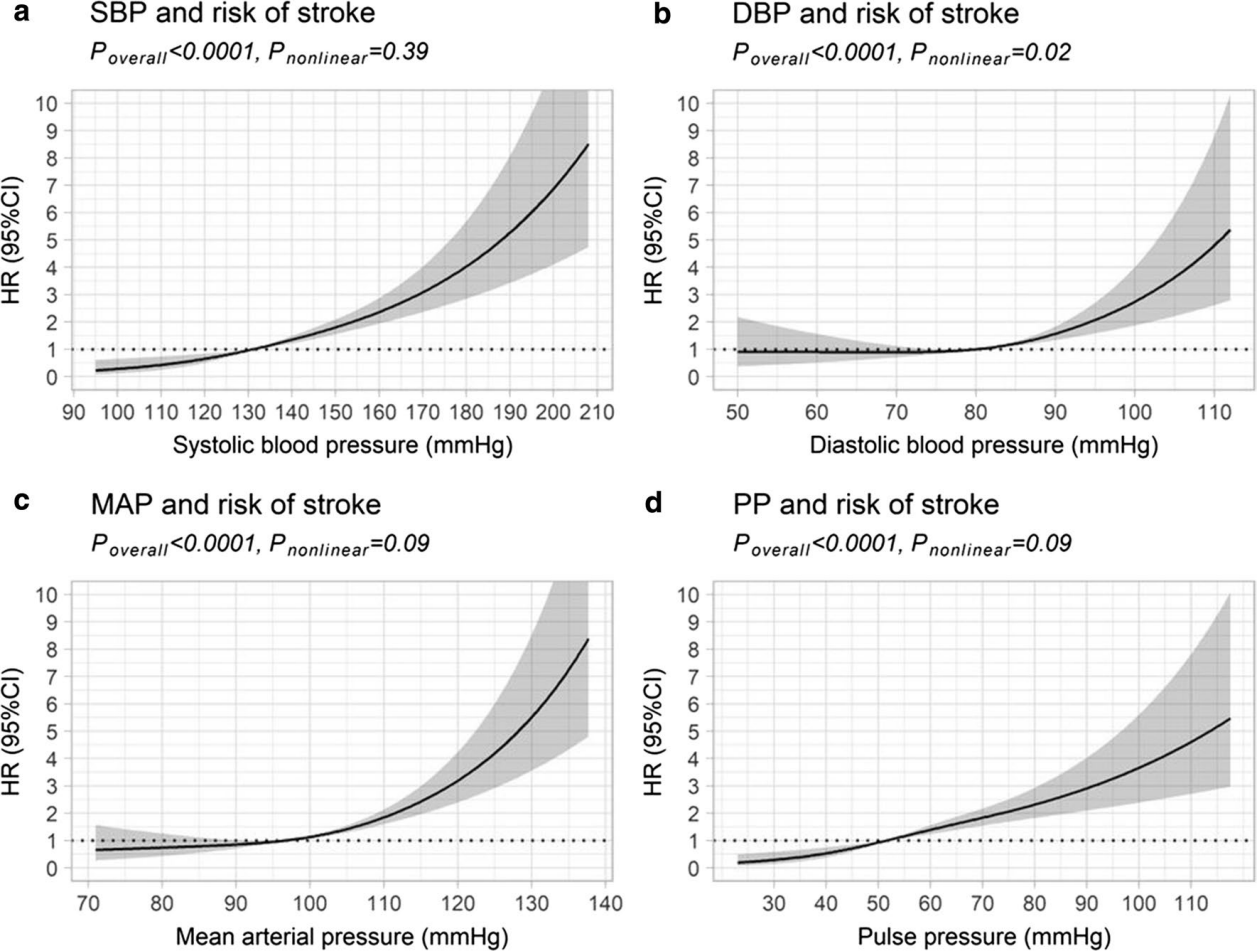
Restricted cubic spline models for any stroke and SBP, DBP, MAP, and PP. Risk of any stroke in relation to **a** systolic blood pressure (SBP), **b** diastolic blood pressure (DBP), **c** mean arterial pressure (MAP), and **d** pulse pressure (PP), estimated using restricted cubic spline models with three knots. The age- and sex-adjusted hazard ratios (HR) are represented by the solid line and the 95% confidence interval (CI) by the shaded area

Cox proportional hazard models using restricted cubic splines were performed to see how the blood pressure variables affected the risk of stroke, without assuming linearity. Figure 1 shows the estimated HRs for stroke for variables SBP, DBP, MAP, and PP. A linear association was found for SBP, MAP, and PP, and the increase in risk was linear across the entire range of blood pressure variable values (Fig. 1). For DBP, however, there was no increase in risk at values below 80 mmHg, after which the risk started to increase (Fig. 1). Comparable results were seen for the stroke subtypes ischemic stroke and hemorrhagic stroke (Additional file 3: Figure S1).

Similar Cox proportional hazards models as for the blood pressure variables were performed for 24-h Na, 24-h K, and Na/K-ratio, and these variables did not increase the risk of stroke and its subgroups in any of the models (data not shown). In the restricted cubic spline analysis for 24-h Na, 24-h K, and Na/K-ratio, no increased risk of any stroke, ischemic stroke, or hemorrhagic stroke was seen for neither low nor high intake (Additional file 4: Figure S2 and Additional file 5: Figure S3).

## Discussion

In this study, we show that all different blood pressure variables, i.e., SBP, DBP, PP, and MAP, independently increased the risk of stroke in individuals with type 1 diabetes. The same except for DBP was seen for ischemic stroke, while SBP, DBP and MAP independently increased the risk of hemorrhagic stroke. The effect of blood pressure variables on the risk of stroke and its subtypes proved to be linear for SBP, MAP, and PP. Notably, no J-shaped association was observed for any blood pressure variable or stroke subtype, neither was sodium or potassium excretion associated with an increased risk of stroke in this study.

### Impact of different blood pressure variables on the risk of stroke

As in the general population [19, 20], SBP seems to be a very strong risk factor for stroke also in type 1 diabetes. SBP remained significant as a risk factor for total stroke and ischemic stroke when compared with the other variables in the Cox regression models. PP is the pulsatile variable of blood pressure and, as such, a marker for large artery stiffness. PP has been shown to increase the risk of stroke in the general population, and the association seems to be linear [2]. The association of DBP and stroke is, however, less clear since this blood pressure variable does not increase with age, instead, it decreases [21], leading to a weaker association with stroke. This also explains the non-linear association with DBP and stroke in the restrictive cubic spline regression models. Rawshani et al. [7] also showed that the association with DBP and stroke is rather weak. MAP, on the other hand, is considered to be the perfusion pressure in organs in the body and is composed of both SBP and DBP [22]. The significant association reflects this for any stroke as well as for both subtypes in the present study. We are not aware of any previous studies on MAP and stroke in type 1 diabetes.

### Blood pressure levels and the risk of stroke

In individuals with type 2 diabetes, the risk of stroke increases after blood pressure exceeds 130/80 mmHg [23]. In our study, the risk of stroke starts to increase at similar DBP levels, while for SBP, linear increase is observed even earlier. In a recent study by Rawshani et al. [7], similar results were seen for SBP, although the risk started to increase for stroke at a slightly higher level compared to our study. The treatment recommendation for blood pressure levels in diabetes is, despite these facts, higher; according to the American Diabetes Association [24], medical treatment of high blood pressure in diabetes should be initiated at blood pressure levels of > 140/90 mmHg, and the treatment goal is a blood pressure of < 140/90 mmHg. However, a recently published guideline by the American Heart Association suggested that blood pressure levels in type 1 diabetes should be lower, recommending initiation of treatment already at blood pressure levels of > 130/80 mmHg, and that the blood pressure target should be < 130/80 mmHg in individuals with diabetes [22]. The results in our study support the new recommendations for blood pressure treatment targets in type 1 diabetes. Furthermore, despite the high prevalence of antihypertensive medication in the participants with stroke blood pressure levels were suboptimal, indicating that a more aggressive approach in treating blood pressure in these individuals should be considered.

### Association of blood pressure variables and stroke

To our surprise, there were no indications of a J-shaped phenomenon in our study for any of the studied blood pressure variables. The risk of stroke increased linearly for all subtypes for SBP, MAP, and PP, while the risk increment was non-linear for DBP. For SBP, this is in line with the study by Rawshani et al. [7], where the association with stroke was also linear. According to Zhao et al. [5], the J-shaped association was found mainly in individuals 30–59 years of age, and this association weakened in individuals older than 60 years. Even though the participants in our study were younger than 60 years, mean age around 50 years, their blood vessels could resemble those of non-diabetic individuals of older age due to the long diabetes duration and, thus, alternating blood glucose levels. In accordance with this, we have earlier shown that the pulse pressure in individuals with type 1 diabetes increases 15 to 20 years earlier than in non-diabetic individuals, suggesting early vascular aging in diabetes [25]. This can be explained by the fact that hyperglycemia causes endothelial dysfunction, leading to diabetic angiopathy and arterial stiffness, and all of these leading to hypertension [26–28]. The same changes take place in normoglycemic persons during aging [29, 30]. On the other hand, apart from hypertension, the risk factors identified for stroke in type 1 diabetes are different compared to the general population [8, 31, 32]. The etiology of stroke is also different. In type 1 diabetes, the majority of strokes are caused by small-vessel disease or stroke of unclear etiology, while cardioembolism is less frequent in young individuals with type 1 diabetes compared with people without diabetes [33]. Carotid stenosis, a marker of large-artery atherosclerosis, is also less common, especially compared to individuals with type 2 diabetes [33]. Subclinical atherosclerosis is, however, known to associate with type 1 diabetes [34], so it might be that some of the strokes classified as unclear etiology could be due to atherosclerosis. In the present study we were unable to assess the etiology of strokes due to variation in and/or lack of etiological assessment performed at the different hospitals during the different time periods.

### Salt intake and stroke

High salt intake, measured as 24-h urinary sodium excretion, increases the risk of cardiovascular disease, mainly stroke [9, 35]. The same can be seen in individuals with chronic kidney disease, for which the risk of stroke increases linearly when urinary sodium excretion increases [36]. A majority of our participants, 61%, with stroke had impaired kidney function, defined as diabetic nephropathy. Yet, we did not find any associations with urinary sodium and potassium excretion and stroke. One possibility for this result could be the low number of individuals tested; we only had information on urinary sodium and potassium excretion available for 115 participants with stroke. Many of these individuals also had micro- or macroalbuminuria, which in some part affect the urinary excretion. On the other hand, no trends towards any associations were seen in the analyses.

### Study limitations

One of the weaknesses of this study was that we only had an office blood pressure measurement from a single time point available, and this could have been measured years before the incident stroke. Likewise, 24-h Na and 24-h K were also measured from a single urine collection. Ambulatory 24-h blood pressure or timed blood pressure measurements during a more extended time would be the best method to determine the impact of blood pressure on the risk of stroke. Unfortunately, in this large study population consisting of over 4000 participants, this was not feasible. Moreover, already with this single office blood pressure, we were able to show an increased risk of stroke in these participants, indicating that this single blood pressure measurement might be a sensitive enough tool to evaluate the risk of stroke years before an event.

Another limitation of this study is the number of stroke cases, particularly that of hemorrhagic strokes. This could affect the impact of sodium and potassium on the risk of stroke, as well as the J-shaped vs linear association between blood pressure levels and stroke risk. On the other hand, in the studies by Cederholm et al. [37, 38], no J-shaped relationships between low SBP and increased stroke risk was found even though the number of individuals was threefold larger in their second study, 13,000 vs 35,000 participants. Further studies on the potential J-shaped association between blood pressure and stroke in individuals with type 1 diabetes are required before any conclusions can be made.

The number of individuals studied is also one of the strengths of this study. Stroke is, fortunately, still a relatively rare complication in this age group. To this date, this is the largest study population of individuals with type 1 diabetes and stroke ascertained from medical files, which allowed us to also perform studies on the subgroups of stroke, ischemic and hemorrhagic stroke. Another strength in our study is the well-characterized population. All of the included strokes were identified from different sources, the information on strokes and their complications were verified from medical records, and all the included strokes were confirmed and classified with the same methods by expert stroke neurologists.

## Conclusions

In conclusion, we show that different blood pressure variables all independently increase the risk of stroke in individuals with type 1 diabetes, and that the risk of any stroke, ischemic stroke, and hemorrhagic stroke increases linearly already at blood pressure levels lower than the current treatment goals. Intensified treatment of blood pressure to target levels should be taken into consideration in these individuals in the attempt of reducing the risk of future strokes.

## Additional files

**Additional file 1: Table S1.** Location of knots and median for each variable in restricted cubic spline analysis.

**Additional file 2: Table S2.** Baseline characteristics of participants with no stroke, any stroke, ischemic stroke, and hemorrhagic stroke during follow-up.

**Additional file 3: Figure S1.** Restricted cubic spline models for ischemic and hemorrhagic stroke and SBP, DBP, MAP, and PP.

**Additional file 4: Figure S2.** Restricted cubic spline models for any stroke and 24-h Na, 24-h K, and Na/K ratio.

**Additional file 5: Figure S3.** Restricted cubic spline models for ischemic and hemorrhagic stroke and 24-h Na, 24-h K, and Na/K ratio.

**Additional file 6: Appendix.** List of the FinnDiane study centers.

## Data Availability

The datasets generated and/or analysed during the current study are not publicly available due the local legislation and the written consents of the FinnDiane study participants, which do not allow sharing individual-level phenotype data.

## Abbreviations

SBP: systolic blood pressure
DBP: diastolic blood pressure
PP: pulse pressure
MAP: mean arterial pressure
24-h Na: 24 h urinary sodium
FinnDiane: The Finnish Diabetic Nephropathy Study
UAER: urinary albumin excretion rate
DN: diabetic nephropathy
SDR: severe diabetic retinopathy
24-h K: 24-h urinary potassium
Na/K ratio: sodium/potassium ratio
HR: hazard ratio
CI: confidence interval
