# Supplementary material for: The role of blood pressure in risk of ischemic and hemorrhagic stroke in type 1 diabetes
Source: Cardiovasc Diabetol. 2019 Jul 9;18:88. doi: 10.1186/s12933-019-0891-4 (PMC6617855; doi:10.1186/s12933-019-0891-4)
Supplement: Supplementary file 1 — Additional file 1: Table S1. Location of knots and median for each variable in restricted cubic spline analysis. [file 12933_2019_891_MOESM1_ESM.docx]

***ADDITIONAL TABLE 1. Location of knots and median for each variable in restricted cubic spline analysis.***

| **Variable** | **1^st^ knot** | **2^nd^ knot (median)** | **3^rd^ knot** |
| --- | --- | --- | --- |
|  |  |  |  |
| Systolic blood pressure | 113 | 131 | 159 |
| Diastolic blood pressure | 67 | 80 | 92 |
| Mean arterial pressure | 84 | 97 | 112 |
| Pulse pressure | 38 | 52 | 75 |
| 24-hour urinary sodium excretion | 74 | 140 | 236 |
| 24-hour urinary potassium excretion | 45 | 83 | 129 |
| Sodium/potassium ratio | 1 | 1.75 | 2.94 |
|  |  |  |  |

Location of knots in restrictive cubic spline models. The 1^st^ knot was placed at the 10^th^ percentile, the 2^nd^ knot was placed at the 50^th^ percentile (median), and the 3^rd^ knot was placed at the 90^th^ percentile for each variable.
