# Supplementary material for: The role of blood pressure in risk of ischemic and hemorrhagic stroke in type 1 diabetes
Source: Cardiovasc Diabetol. 2019 Jul 9;18:88. doi: 10.1186/s12933-019-0891-4 (PMC6617855; doi:10.1186/s12933-019-0891-4)
Supplement: Supplementary file 2 — Additional file 2: Table S2. Baseline characteristics of participants with no stroke, any stroke, ischemic stroke, and hemorrhagic stroke during follow-up. [file 12933_2019_891_MOESM2_ESM.docx]

***ADDITIONAL TABLE 2. Baseline characteristics of participants with no stroke, any stroke, ischemic stroke, and hemorrhagic stroke during follow-up.***

| BASELINE DATA | *No*  *Stroke* | *Any*  *Stroke* | *Ischemic*  *Stroke* | *Hemorrhagic*  *Stroke* |
| --- | --- | --- | --- | --- |
| *n* | 3,903 | 202 | 145 | 57 |
| Age at onset of diabetes (years) | 14.2 (9.3-22.5) | 13.1 (8.1-21.0) | 15.0 (9.3-23.2) | 12.2 (6.1-22.8)* |
| BMI (kg/m^2^) | 24.9 ± 3.6 | 25.0 ± 3.8 | 25.4 ± 3.9 | 24.0 ± 3.3* |
| Waist circumference (cm) | 85.3 ± 11.1 | 88.7 ± 12.3* | 89.2 ± 11.9 * | 87.6 ± 13.3 |
| Lipids and lipoproteins |  |  |  |  |
| Total cholesterol (mmol/l) | 4.9 ± 1.0 | 5.3 ± 1.1* | 5.4 ± 1.1* | 5.3 ± 1.3* |
| LDL cholesterol (mmol/l) | 3.0 ± 0.9 | 3.3 ± 1.0* | 3.4 ± 1.0* | 3.2 ± 1.2 |
| HDL cholesterol (mmol/l) | 1.3 ± 0.4 | 1.3 ± 0.4 | 1.3 ± 0.4* | 1.4 ± 0.4 |
| Triglycerides (mmol/l) | 1.02 (0.77-1.45) | 1.31 (0.95-1.85)* | 1.22 (0.94-1.89)* | 1.20 (0.92-1.71)* |
| Glucose control |  |  |  |  |
| HbA_1c_ (%) | 8.4 ± 1.5 | 8.9 ± 1.3* | 8.9 ± 1.3* | 8.7 ± 1.5 |
| HbA_1c_ (mmol/mol) | 69 ± 17 | 73 ± 15* | 74 ± 14* | 72 ± 17 |
| Insulin dose (IU/kg) | 0.70 ± 0.27 | 0.67 ± 0.24 | 0.66 ± 0.21* | 0.70 ± 0.29 |
| Micro- and macrovascular complications |  |  |  |  |
| Urinary AER (mg/24h) | 11.3 (6.4-37.8) | 82.1 (14.0-573.6)* | 69.6 (15.5-372.4)* | 37.4 (5.7-130.4)* |
| Diabetic nephropathy (%) | 20 | 62* | 61* | 63* |
| End-stage renal disease (%) | 6 | 27* | 22* | 42* |
| Severe diabetic retinopathy (%) | 31 | 75* | 75* | 77* |
| Coronary heart disease (%) | 5 | 13* | 16* | 5 |
| Medication and smoking |  |  |  |  |
| Lipid-lowering medication (%) | 11 | 25* | 23* | 31* |
| Aspirin (%) | 12 | 28* | 29* | 27* |
| Warfarin (%) | 0 | 1 | 0 | 2 |
| Current or history of smoking (%) | 45 | 62* | 67* | 49 |

Data are presented as means ± standard deviation, median with interquartile range, or number of cases (%).

*=P<0.05 compared to no stroke. BMI = body mass index, AER= albumin excretion rate
