# Supplementary material for: The role of blood pressure in risk of ischemic and hemorrhagic stroke in type 1 diabetes
Source: Cardiovasc Diabetol. 2019 Jul 9;18:88. doi: 10.1186/s12933-019-0891-4 (PMC6617855; doi:10.1186/s12933-019-0891-4)
Supplement: Supplementary file 3 — Additional file 3: Figure S1. Restricted cubic spline models for ischemic and hemorrhagic stroke and SBP, DBP, MAP, and PP. [file 12933_2019_891_MOESM3_ESM.docx]

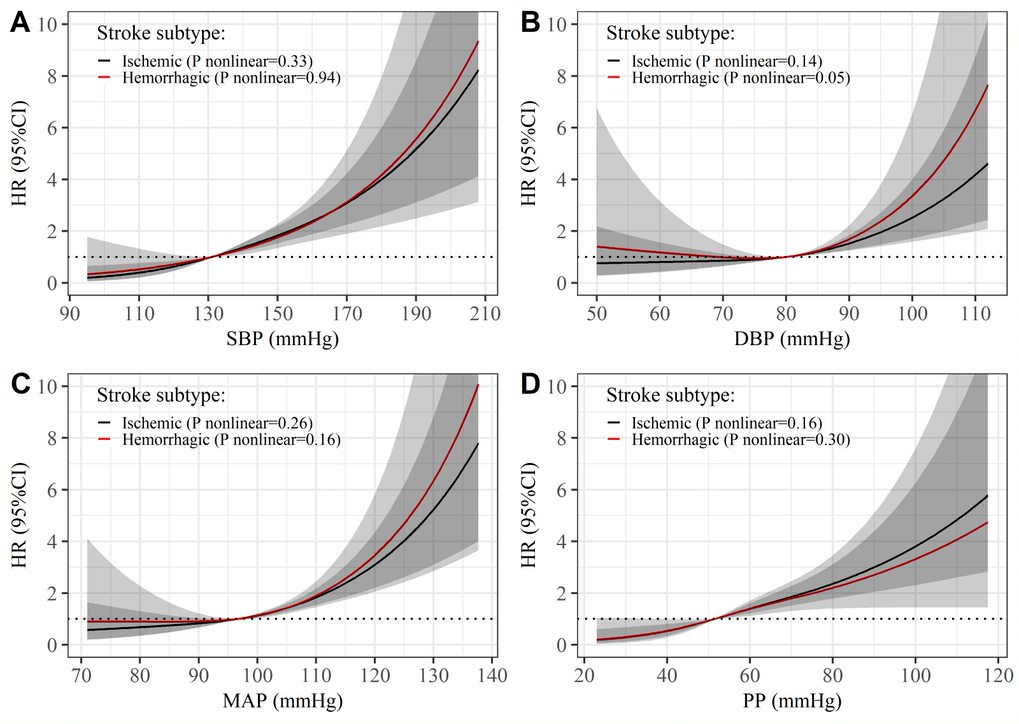


Additional Figure 1. Restricted cubic spline models for ischemic and hemorrhagic stroke and SBP, DBP, MAP, and PP. Risk of ischemic and hemorrhagic stroke in relation to A. Systolic blood pressure (SBP), B. Diastolic blood pressure (DBP), C. Mean arterial pressure (MAP), and D. Pulse pressure (PP), estimated using restricted cubic spline models with three knots. The age- and sex-adjusted hazard ratios (HR) are represented by the solid line and the 95% confidence interval (CI) by the shaded area.
