# Supplementary material for: The role of blood pressure in risk of ischemic and hemorrhagic stroke in type 1 diabetes
Source: Cardiovasc Diabetol. 2019 Jul 9;18:88. doi: 10.1186/s12933-019-0891-4 (PMC6617855; doi:10.1186/s12933-019-0891-4)
Supplement: Supplementary file 4 — Additional file 4: Figure S2. Restricted cubic spline models for any stroke and 24-h Na, 24-h K, and Na/K ratio. [file 12933_2019_891_MOESM4_ESM.docx]

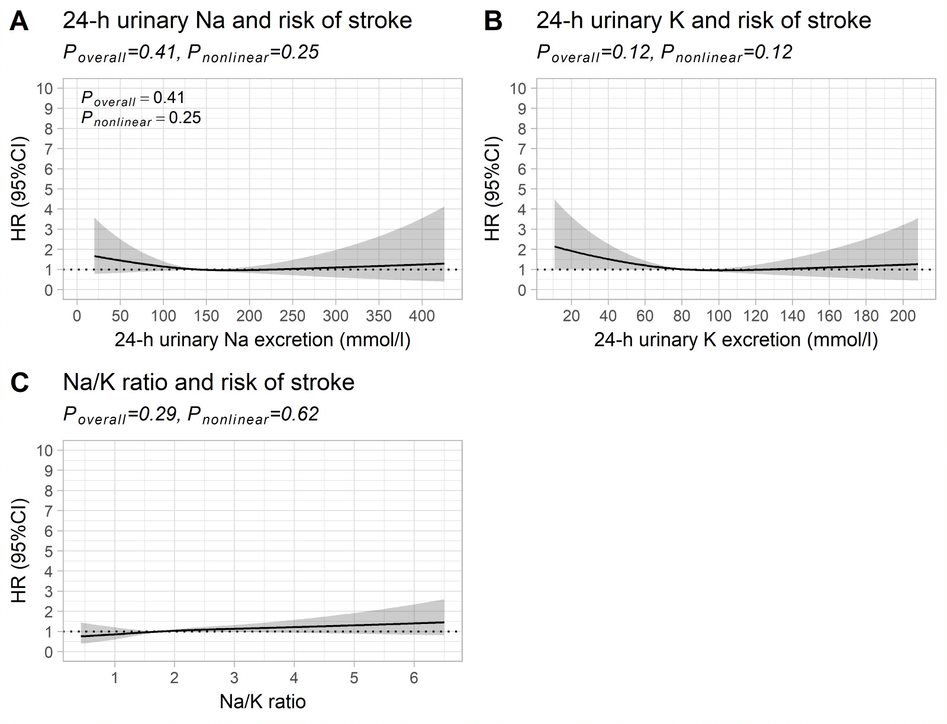


Additional Figure 2. Restricted cubic spline models for any stroke and 24-h Na, 24-h K, and Na/K ratio. Risk of any stroke in relation to A. 24-hour urinary sodium excretion (24-h Na), B. 24-hour urinary potassium excretion (24-h K) C. Sodium/potassium ratio (Na/K ratio), estimated using restricted cubic spline models with three knots. The age- and sex-adjusted hazard ratios (HR) are represented by the solid line and the 95% confidence interval (CI) by the shaded area.
